# Medical machine learning operations: a framework to facilitate clinical AI development and deployment in radiology

**DOI:** 10.1007/s00330-025-11654-6

**Published:** 2025-05-08

**Authors:** José Guilherme de Almeida, Christina Messiou, Sam J. Withey, Celso Matos, Dow-Mu Koh, Nickolas Papanikolaou

**Affiliations:** 1 Champalimaud Foundation, Lisbon, Portugal; 2 Department of Radiology, Royal Marsden Hospital, Sutton, UK

**Keywords:** Artificial intelligence, Medical informatics applications, Decision making, Computer-assisted

## Abstract

**Abstract:**

The integration of machine-learning technologies into radiology practice has the potential to significantly enhance diagnostic workflows and patient care. However, the successful deployment and maintenance of medical machine-learning (MedML) systems in radiology requires robust operational frameworks. Medical machine-learning operations (MedMLOps) offer a structured approach ensuring persistent MedML reliability, safety, and clinical relevance. MedML systems are increasingly employed to analyse sensitive clinical and radiological data, which continuously changes due to advancements in data acquisition and model development. These systems can alleviate the workload of radiologists by streamlining diagnostic tasks, such as image interpretation and triage. MedMLOps ensures that such systems stay accurate and dependable by facilitating continuous performance monitoring, systematic validation, and simplified model maintenance—all critical to maintaining trust in machine-learning-driven diagnostics. Furthermore, MedMLOps aligns with established principles of patient data protection and regulatory compliance, including recent developments in the European Union, emphasising transparency, documentation, and safe model retraining. This enables radiologists to implement modern machine-learning tools with control and oversight at the forefront, ensuring reliable model performance within the dynamic context of clinical practice. MedMLOps empowers radiologists to deliver consistent, high-quality care with confidence, ensuring that MedML systems stay aligned with evolving medical standards and patient needs. MedMLOps can assist multiple stakeholders in radiology by ensuring models are available, continuously monitored and easy to use and maintain while preserving patient privacy. MedMLOps can better serve patients by facilitating the clinical implementation of cutting-edge MedML and clinicians by ensuring that MedML models are only utilised when they are performing as expected.

**Key Points:**

***Question*** *MedML applications are becoming increasingly adopted in clinics, but the necessary infrastructure to sustain these applications is currently not well-defined*.

***Findings*** *Adapting machine learning operations concepts enhances MedML ecosystems by improving interoperability, automating monitoring/validation, and reducing deployment burdens on clinicians and medical informaticians*.

***Clinical relevance*** *Implementing these solutions eases the faster and safer adoption of advanced MedML models, ensuring consistent performance while reducing workload for clinicians, benefiting patient care through streamlined diagnostic workflows*.

## Introduction

Machine-learning (ML) models have been at the forefront of recent advances in computationally-assisted diagnosis systems in medicine and, particularly, in radiology [1, 2]. These medical machine learning (MedML) models are mathematical and computational constructs capable of generating a diagnosis or computing a risk score from patient data. These models have demonstrated significant potential in various radiology applications, including disease management aspects such as screening [3], diagnosis, prognosis and treatment monitoring [4]. They show potential applications for early detection [5] and incidental findings [6, 7]. A recent preprint suggested MedML models could also be used to triage patients, recommending manual diagnosis only when the MedML system is uncertain [8]. Other MedML systems can improve the workflow of radiologists. Deep-learning-assisted image reconstruction of CT [9] and MRI images [10, 11] can reduce acquisition times. Additionally, automatic segmentation [12–14] can accelerate diagnosis by facilitating anatomical volumes quantification, or be applied for radiotherapy treatment planning [15, 16]. With medical professionals, MedML has the potential to improve routine medical exam accuracy [3, 17] while lowering costs and reducing workload [15, 18–20].

**Fig. 1:**
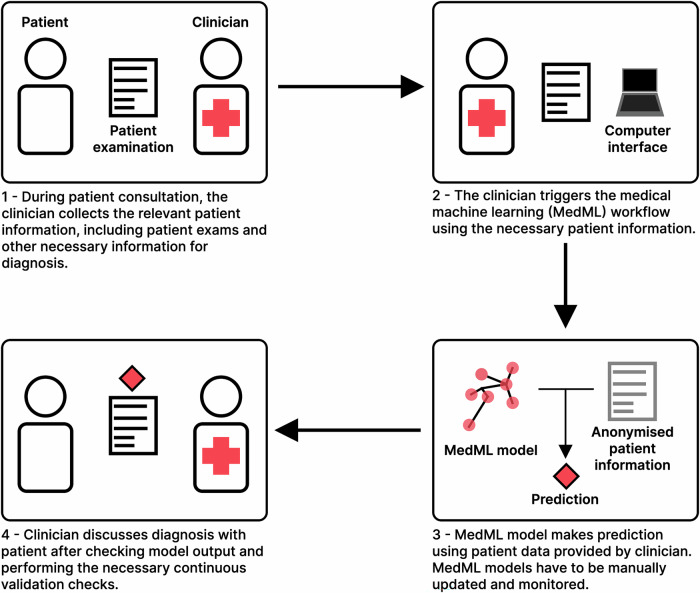
Medical machine learning workflow with MedML. The patient sees their clinician as usual and undergoes a given number of exams. The medical doctor uses these exams to run a MedML workflow, which produces a prediction. The clinician then confirms and discusses the prediction from the model

A typical MedML workflow in radiology (Fig. 1) involves:

i. Data collection after patient consultation and examination.
ii. Prediction (or inference) after de-identification (if the prediction happens outside of the medical centre) using a trained and validated model.
iii. Confirmation of the results of the model output by a trained medical expert after re-identification (if necessary).
iv. Discussion of results with the patient or application of the results for patient management.

We refer to the orchestration of this workflow as a MedML system, integrating one or more MedML models to deliver clinical predictions.

## Hurdles in medical machine-learning (MedML) deployment

While promising, the potential of MedML is hard to realise. A 2024 report surveying 34 medical centres showed that while 73% wanted to deploy clinical artificial intelligence (AI) systems, only 16% had governance policies on AI and data usage [21]. A study on federated learning—where a model is concurrently optimised across medical centres without centralising data—highlighted the necessity for coordinated computational infrastructure if hospitals are unable to externalise data during prediction [22]. Additionally, updating digital medical practices can be time-consuming [23]. Implementing AI tools in clinical practice was complicated as it requires routine performance assessments and clear articulation of requirements among various stakeholders, including medical, technical, and financial teams [24]. A survey of health professionals highlighted the need for alternative and innovative approaches facilitating continuous model assessment [25]. Studies also show that a significant portion of healthcare workers and students lack adequate education on MedML systems [26–31]. A qualitative assessment on the adoption of a MedML system reflected similar findings [32].

Furthermore, ML model deployment is highly experimental, as noted in a survey of ML engineers [33]. When translating models from data science to usable models in the real world, ML engineers actively experiment to understand how the model behaves and how continuous validation/tuning impacts performance. One respondent provided an illustrative quote—“We don’t have a good idea of how the model is going to behave in production until production.” Finally, ML scholarship tends to exaggerate claims and focus on irrelevant validations, making research and public adoption more complicated by reducing reproducibility and creating unrealistic expectations [34, 35].

The issues above are not unique to radiology MedML but are compounded by working with clinical images and data or aggravated by the medical context. Medical data, which is formatted and stored differently depending on hospital-specific medical database systems, has added legal/ethical constraints concerning strict patient confidentiality and anonymity. This forestalls the development of consistent cross-institutional pipelines which are performant and capable of ensuring patient privacy, complicating automated continuous validation. Consequently, continuous data collection and annotation are hindered, making continuous monitoring, validation and (re)training more complicated. This is crucial as changes to protocol and scanner manufacturer [14, 36–39] can affect performance. While speculative, model retraining can become necessary in MedML systems as ML model performance degrades with time [40], an effect also observable in MedML [41, 42].

Here, we do not attempt to claim that clinical institutions are incapable of implementing MedML systems. Instead, we offer a framework—outlined below—which not only facilitates the deployment of MedML systems in radiology practice but also benefits medical, technical, legal, and financial stakeholders. Indeed, no commercial MedML solutions implement continuous validation strategies.

## The growing field of machine-learning operations (MLOps)

To address issues affecting the development and deployment of ML models, the field of MLOps has become increasingly popular. MLOps applies software development operation practices (DevOps; Table 1) to ML development. MLOps comes with additional requirements: the development of data models (ways of automatically checking and cleaning data), continuous monitoring/validation (to ensure model performance does not drop as time progresses), and, sometimes, automated model retraining (if performance drops below a given threshold). Many companies utilise MLOps frameworks to automate data collection and model serving, training and validation in fields such as banking (for card fraud detection, loan approval or credit scoring), retail (for product recommendation or automatic visual recognition of cosmetic products) and deliveries (forecasting the necessary delivery employees at specific times and dynamic pricing) [43]. Importantly, a survey of over 300 data scientists highlighted how MLOps is crucial for applications where organisations are interested in training, validating, monitoring or deploying multiple models [44], as is the case with MedML, particularly in radiology.

**Table 1:** Definition of software development operations (DevOps)

Definition	Who does it	Key aspects
DevOps can be loosely defined as the practice of facilitating the collaboration between software development teams and information technology/deployment teams [129].	Software development and deployment are typically performed by different teams which prioritise different aspects (this extends to ML [45]), but a sizable fraction of individuals accumulate both roles [34, 41]. For instance, ML model development teams may focus on data preparation and model training/optimisation, whereas ML model deployment teams are concerned with distributing this model to end users and ensuring that data and inference operations are coordinated.	A key aspect of DevOps is continuous integration and continuous deployment (CI/CD), which consists of a software development paradigm focusing on quickly and seamlessly integrating frequent changes to software programmes. This requires additional safety barriers preventing incorrect changes from being exposed to end users, making unit testing and collaborative code reviews essential to the correct functioning of CI/CD frameworks.

However, implementing these platforms is not straightforward. An exposition of two real-world cases focusing on data integration and AI/ML system scaling highlights hurdles present in the deployment and scaling of MLOps platforms. The first case describes how a software product providing data-driven patient-level risks related to hip and knee joint replacement surgery managed to overcome challenges associated with data integration and harmonisation. The second case focuses on how a government-initiated programme scaled a software responsible for matching citizens with the best possible public services [45]. The issues they run into are common to large-scale data-driven approaches, and can be related to modern problems in radiology MedML:

- Data integration (or harmonisation) was laborious during deployment as this required integrating data from multiple sources/formats in a data lake (a centralised data repository with a uniform format), only then making ML model training/monitoring possible. In radiology, different vendors/models have specific tags referring to specific parameters necessary for data pre-processing. Additionally, data harmonisation between medical centres is challenging due to scanner-specific artefacts or shifts in the population being assessed.
- Scaling challenges: the example highlighted difficulties in scaling an application designed for individual users in Finland. Since it relied on users providing their own data, encouraging people to share data and verify sample authenticity were major hurdles. For instance, in medical ML, collecting informed consent can be tricky, but telemedicine methods may help solve this [46]. In radiology, informed consent collection is crucial for prospective model validation, but few centres have simple, electronic methods enabling large-scale collection.
- Model deployment: during model deployment, combining data sources for model training becomes necessary, requiring either harmonising data from multiple sources or integrating different models. The former additionally requires protocols for data sharing, while the latter may increase the number of faults in ML models [47]. In radiology, there are already multiple platforms for model deployment/orchestration, but few offer holistic integration between different models.
- Monitoring issues: monitoring also became complicated—the burden of monitoring and validating predictions was on end users, leading to missing validation or inaccurate data. To address this in medical centres, automating data de-identification, collection, and organisation is crucial. In radiology, prospective data collection is essential for model validation, but collecting data is mostly performed “manually”: radiologists dedicate time and energy to gather data across institution-specific databases.

These issues are not particular to MLOps but are illustrative of issues which arise when scaling applications. Nonetheless, and similarly to DevOps [48], other issues such as data quality, compute and model complexity can arise during MLOps deployment [49].

## Medical machine-learning operations (MedMLOps)

**Fig. 2:**
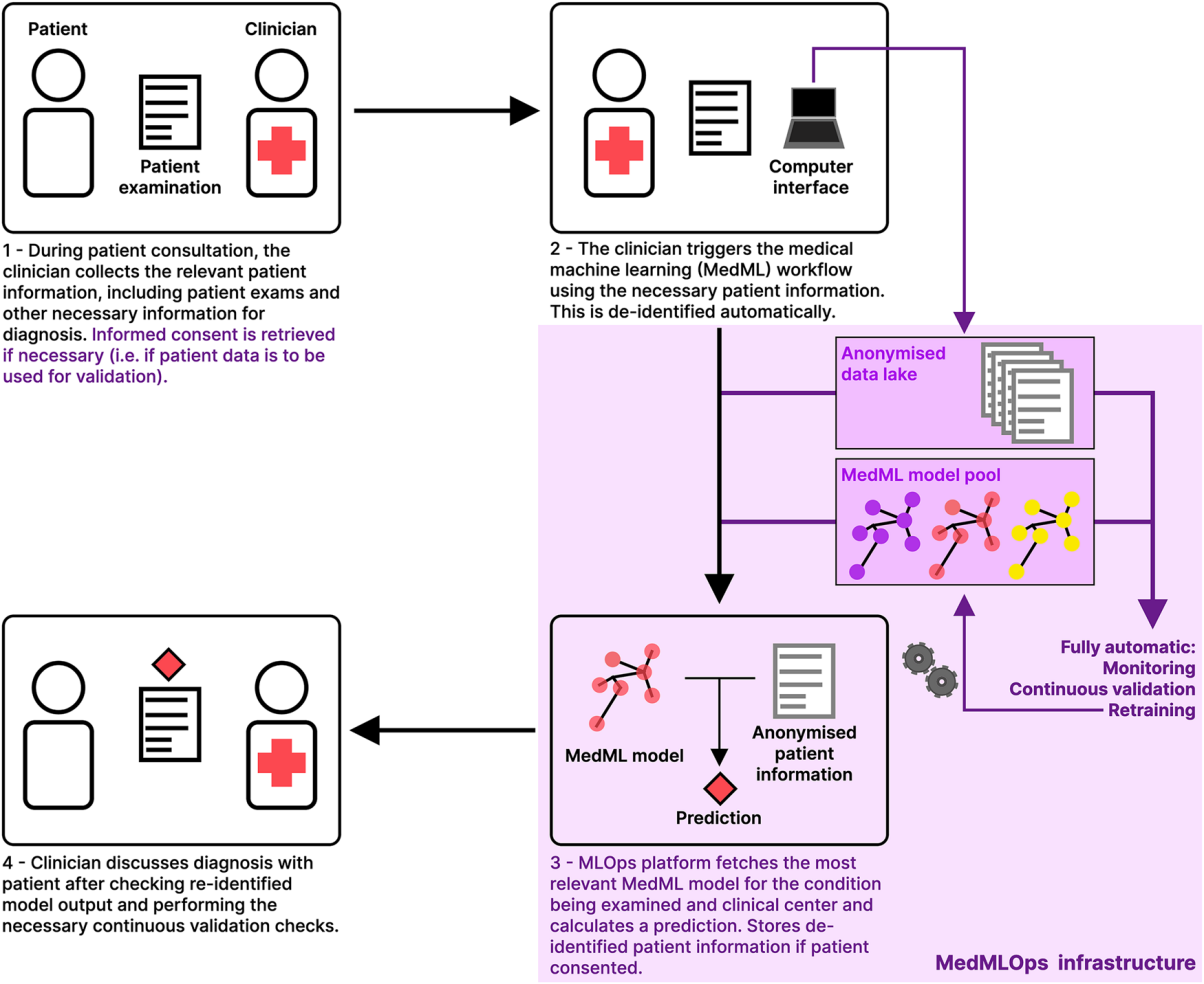
Medical machine learning workflow with MedMLOps. The workflow for the patient and clinician is identical to that of a typical MedML workflow. However, the MedML pipeline, through the MedMLOps infrastructure, orchestrates anonymisation, model selection, validation, inference, and optional data storage, which will be used to continuously validate and retrain the model. The key differences between the traditional MedML workflow and the MedMLOps workflow are highlighted in pink and purple colours

**Fig. 3:**
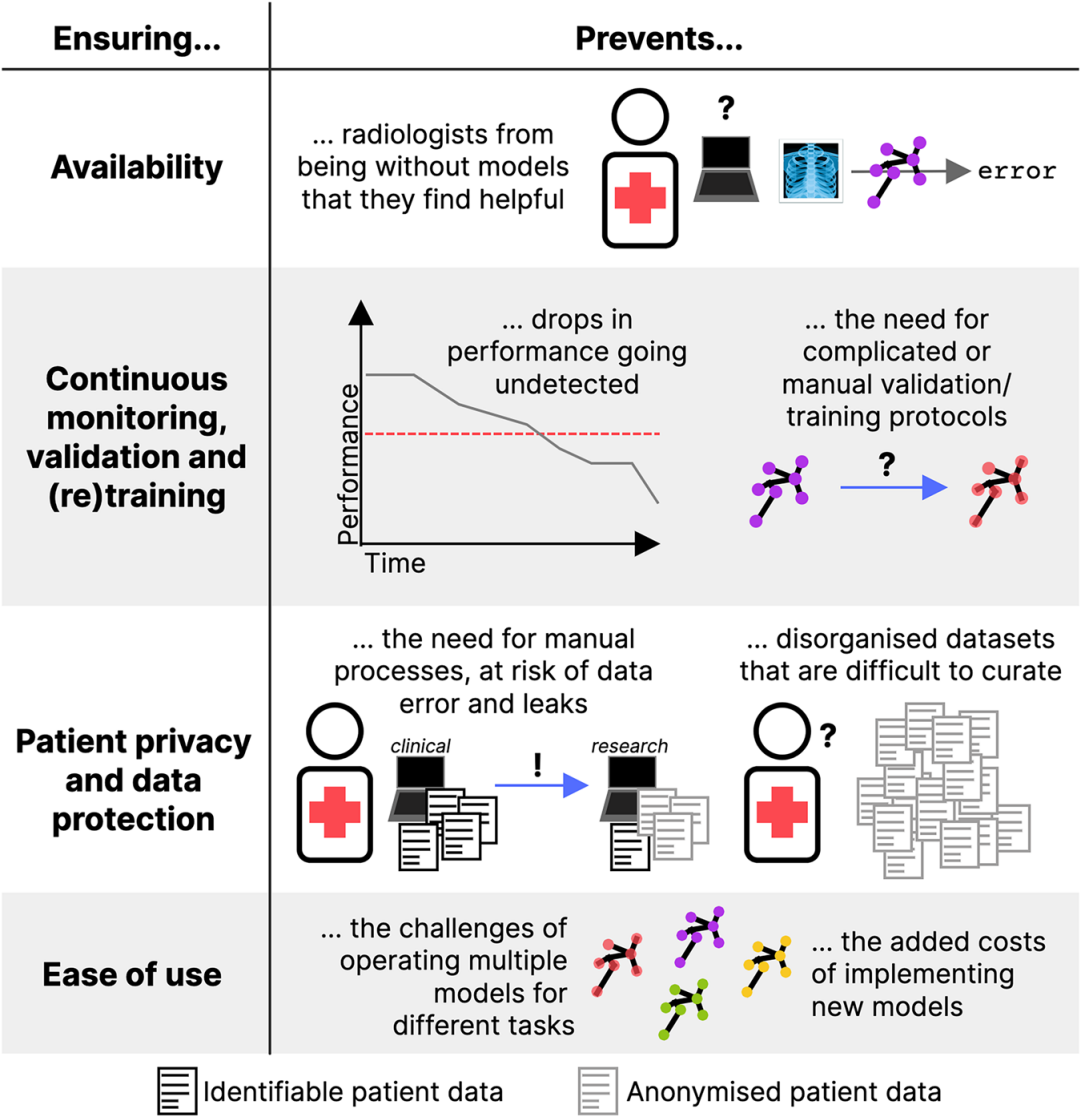
How the four pillars suggested in this work can solve some issues in MedML applications. Each of the four pillars focuses on a specific aspect of medical machine learning model deployment: (i) ensuring availability leads to fault-tolerance and robustness to malicious actors, (ii) ensuring continuous monitoring, validation and (re)training leads to performance which is consistently good and facilitates model retraining, (iii) ensuring patient privacy and data protection leads to automated protocols for data de-identification, transfer and curation/organisation, and (iv) ease of use ensures that models are more interchangeable and interoperable

Considering the technical challenges in MedML development, we suggest MLOps as a grounded perspective to address some problems associated with deploying MedML. We refer to the integration of MedML with MLOps as MedMLOps (Medical Machine Learning Operations) and suggest four distinct pillars: i) availability, ii) continuous monitoring, validation and (re)training, iii) patieent privacy and data protection, and iv) ease of use^1^ (Fig. 2). Through this article, we show how these four pillars (Fig. 3) are crucial for MedML systems, starting with short, focused descriptions of each to better illustrate their importance from a clinical radiology perspective:

- Availability. MedMLOPs systems ensure that radiologists can always use the same models, guaranteeing that patients can always benefit from consistent clinical services.
- Continuous monitoring and validation ensure that models do not unexpectedly fail after radiologists become accustomed to them. Importantly, having automatic frameworks to detect when MedML systems fail reduces the risk of an underperforming MedML system becoming a liability to medical centres and patients alike.
- To ensure patient privacy and data protection, automated informed consent collection and data de-identification, curation and storage mechanisms are necessary. A MedMLOps framework reduces the risk of exposing untrusted parties to patient data: this may result from mistakes during data retrieval/de-identification from picture archiving and communication system (PACS). MedMLOps also guarantees easily retrievable and de-identifiable data if researchers and clinicians hope to further develop MedML models.
- Finally, ease of use concerns both how radiologists interact with these systems and ease of implementation: with standardised MedMLops systems and protocols, the burden of changing between MedML vendors/products is reduced.

While MLOps is not the sole paradigm offering these four pillars. It is especially positioned as a competent, varied and growingly popular field capable of addressing problems associated with MedML model development and deployment while preserving clinician, physician and patient experience.

Before continuing, we note the difference between diagnostic and forecasting approaches. Diagnostic models produce predictions which can be quickly verified by experts. Forecasting models are risk models, producing a quantity which correlates with the probability of a patient developing a condition in the future.

### Availability

To ensure MedML model utilisation, availability is essential. Two aspects of many MLOps frameworks ensure this—fault tolerance and scalability. These depend on two prediction paradigms: “model-to-data” (MTD) or “data-to-model” (DTM) [51]. In MTD, the model is accessed (or served) locally when prediction becomes necessary. DTM, on the other hand, involves moving data to a remote server where predictions are performed and then returned. While the former has the advantage of preserving privacy, it requires dedicated computational resources in medical institutions. The latter, on the other hand, requires only that hospitals have an internet connection but exposes patient data to man-in-the-middle attacks (where malicious actors access data during data transfer) [52] or other data leaks. These can facilitate data theft or data tampering, causing erroneous predictions [53, 54].

Fault tolerance consists of mechanisms that restart system components when they crash or become unresponsive, ensuring service availability. Frameworks such as Kubernetes^2^ are capable of providing this through the cloud and in edge computing scenarios (i.e. when computational resources are scarce) [55]. Scalability is the automatic capability of allocating resources as computational demand increases. In practical terms, while MTD is based mostly on transferring models to medical centres and requires little scalability, DTM requires a persistent network connection, as well as the capacity to scale the number of predictions it can offer as demand increases. Allocating excessive resources becomes costly, while allocating too few can lead to models with unpredictable availability.

Ultimately, availability is considered important in digital medical applications [56, 57], and its importance should only increase as medical systems become more dependent on digital technologies.

### Continuous monitoring, validation and (re)training

Model performance slowly degrades with time [40], obviating the need for continuous monitoring and validation. However, a concrete problem arises—when delegating decisions to MedML, how is validation possible? Assessing the veracity of model outputs requires ground truth annotations. Similarly, training MedML models requires human-annotated data. However, if ground truths are generated by a MedML model, this can lead to model collapse, which happens when models are trained on data or annotations generated by other models (i.e. synthetic data) and leads to underperforming models. While creative methodologies can prevent this [58], it affects different models to different extents [59].

Addressing this at the local level requires considering diagnostic models separately from forecasting models. Diagnostic model validation can be done by considering MedML models as second readers, a well-known approach in radiological screening and particularly useful for less experienced clinicians [17, 20, 60, 61]. If the model performs well, this process could require the manual annotation of a small random subset of cases to estimate performance drops (i.e. model performance falling below a performance threshold defined internally or by vendors). For forecasting models, however, continuous validation becomes paradoxical if these models replace proper follow-ups and diagnoses. Consequently, forecasting models should be considered risk models—a high-risk prediction should lead to a confirmatory or more frequent follow-up. This creates the data necessary to continuously evaluate these approaches, with the caveat that this validation protocol only assesses the true positive rate (or sensitivity) of a model.

Effective continuous validation should span multiple centres. This can be integrated into radiology workflows with MedMLOps as an additional service, requesting annotations for randomly selected cases. This enables the collection of patient consent and facilitates long-term performance record-keeping. This might increase the workload for medical doctors as it will require introducing data into MedML platforms after prediction (especially considering forecasting models). However, the time- and cost-savings afforded by MedML models [62] can compensate for this, and companies should compensate hospitals and individuals for the added labour. Additionally, automated data de-identification, collection, and organisation processes (as noted below), can facilitate this. Patient agency should also be prioritised: informed consent should explicitly state whether their data will be used to validate commercial models.

After performance drops are detected—typically due to data drifts, characterised by changes in the distribution of the underlying patient population data [63] — retraining can be automatically or manually triggered [64]. This requires additional considerations. As noted, patient data must be retrieved, and patient consent should be collected for randomised subsets of cases. This then requires a careful balance of older data (annotated under controlled circumstances) and more recent data (closer to real-world data) for model retraining [65].

A common approach to update models is fine-tuning with recent data [66]. However, this can lead to “catastrophic forgetting”: subsequent retraining processes with data too dissimilar to the original training data lead to performance drops [67]. Some approaches mitigate this [68, 69], but continuous model assessment is still essential. Continuous learning approaches have been implemented in radiology MedML [70], but they may be hampered by catastrophic forgetting due to the nature of medical practice (Table 2). Different versions of the same model may be necessary at the same time, all of which require continuous validation and adaptation as necessary. While currently not possible from a regulatory perspective, a “pool of models”, automatically selected to best fit each centre, may help avoid scenarios of catastrophic forgetting. The continuous integration of MLOps, combined with model and data versioning tools (used to track differences in versions of the same data and model) [71], is especially fitted to dynamically distribute these models. However, this iterative branching of models may lead to a plethora of models, which is hard to manage and monitor—a careful balance must be struck between “models that produce an output for everyone” and “models that are clinically useful”.

**Table 2:** Speculative example of catastrophic forgetting

Setting	Two hospitals—HA and HB—are using the same version of a MedML model provided by the same company.
Problem	While both used similar scanners for several years, provided by the same vendor, HA recently changed to a different scanner vendor. As expected [14, 37], this led to a drop in performance, triggering the automated retraining of the model.
Outcome	This automatic retraining led to a model which performs well on HA. However, the performance for HB has now dropped due to catastrophic forgetting—while being fitted to new data, the model now underperforms on data more similar to its older distribution. A careful decision is now necessary—should different models be adapted and distributed to HA and HB? Or should HB terminate its use of the MedML model, and does it have the necessary contingencies to do so?

Finally, performance drops detected during continuous validation may not necessarily lead to retraining. Indeed, without the appropriate training data (due to ethical constraints or low patient volume), it may be the case that radiology departments have to abandon MedML models altogether. Hospitals and MedML vendors should keep this in mind and plan accordingly. In the end, all stakeholders should prioritise patient safety and care, and MedML models which underperform are directly at odds with this.

### Patient privacy and data protection

Thanks to the General Data Protection Regulation (GDPR), patient privacy became a more prominent concern as violations became liabilities for companies and research centres. Through GDPR, safeguards for patient data increased or became more evident: data de-identification and ethical approval/institutional review board waivers gained visibility, patients became capable of opting out from databases, and concerns with data governance (how data is stored and distributed) increased [72]. Such regulations are more cumbersome and require allocating implementation funds across medical centres [73]. However, they have little impact on biomedical research [74].

The “Regulation of the European Parliament and the Council Laying down harmonised rules on artificial intelligence (Artificial Intelligence Act) and amending certain union legislative acts”—or, colloquially, the EU Artificial Intelligence Act (EUAI)—brought additional regulations concerning patient privacy and how MedML models should behave and be utilised [75]. Particularly, the EUAI considers medical applications to belong to the high-risk category, implying that MedML systems must follow a set of requirements [75, 76]:

- Well-defined scope—high-risk applications must have a specific use and documentation outlining the expected use cases and outcomes, as well as mitigation strategies for potential and identifiable misuse.
- Representative and documented training data—models must be trained with representative and well-documented training data, which is accessible to monitoring agencies.
- Human oversight—AI systems must be designed to permit not only human oversight but also overriding decisions made by them.
- Expected lifetime of the model.
- Computational requirements.

The EUAI also alludes to continuous model monitoring, validation and (re)training. Indeed, it is possible to modify high-risk AI applications if the accompanying documentation concerning their scope and training data is adequately updated [75]. Finally, these requirements, together with GDPR, can block careless implementations of commercial large language models (LLMs) in the clinic. LLMs have ill-defined scopes (i.e. predicting the next word or set of characters or producing text vaguely described as “helpful” or “harmless” [77]) and are trained using undocumented data (making their potential harm hard to quantify without adequate benchmarking [78]). Additionally, these LLMs serve as application programming interfaces (APIs)—which externalise sensitive patient information—making their use hard to justify considering recent EU regulation. Despite these concerns, plentiful scholarship has been produced using commercial LLMs in radiology, from interpretation and diagnosis [79, 80] to error detection in radiology reports [81].

Ethical and legal considerations mandate that patient privacy be central to MedML, especially with large, multi-centric datasets [82]. Consequently, privacy-preserving ML models are gaining importance [83], integrating privacy into training and prediction. This often involves reversible cryptographic methods [84, 85], but scalability remains a challenge [86].

Training with data from multiple centres raises privacy concerns due to increased data breach potential. Large, safe, de-identified centralised repositories [87] and privacy-preserving approaches [88] offer a solution, but ethical issues may impede this. Decentralised learning is gaining traction in medicine, with promising outcomes in radiology [89–91] via federated [92] and swarm learning [89].

MLOps does not ensure patient privacy per se. However, a MedMLOps standard can equip MedML platforms with tools for automated patient anonymisation/de-identification and efficient, anonymised data storage. Furthermore, it can support robust model handling and serving, incorporating predictions into interpretable reports for clinician validation. Image de-identification tools (for modalities which write patient identifiers into the image itself) can also be incorporated into the MedML toolbox.

A MedMLOps standard will enable decentralised interactions with medical databases and facilitate opt-out processes. Indeed, keeping opt-out as the norm and informing patients, facilitates retrospective and consensual data reuse [93, 94]. By extending data ownership schemes, this can better serve end-users. Electronic health records (EHR) are a complex ecosystem, even for clinicians, and their centralised storage can lead to data theft or low availability [95, 96]. Different models of data ownership exist, some placing a heavier focus on patient rights. Others focus on quickly using medical data. While patient data ownership is infrequently discussed in the big data literature [97], some recent medical data storage/sharing paradigms regard patients as legitimate owners of their EHR [96].

### Ease of use

MedML usability affects how clinicians interact with it. MedMLOps enhances these interactions by improving MedML system development and deployment. In Table 3 we highlight this from the perspective of radiology departments hoping to develop, validate or retrain MedML models. In this table, we cover how data collection, data curation and cleaning, model training, validation and model retraining are simplified by MedMLOps. For developers, however, usability relies on how data interacts with APIs [98].

**Table 3:** How MedMLOps can change the radiology workflow for MedML development, validation and retraining at individual institutions. Importantly, these changes are considered for medical centres which have already implemented a MedMLOps system

Process	Without MedMLOps	With MedMLOps
Preliminaries	Medical professionals and researchers identify a relevant research question and what kinds of data will be required.	
Data collection	Clinicians and/or researchers localise specific types of data in PACS and EHR systems by hand or using minimal automations based on series descriptions and image types.	Data is automatically collected and stored in a data lake following appropriate informed consent and anonymisation or pseudonymisation by orchestrating communications with PACS and HER systems. An automated system of telemedicine informed can be used to guarantee that physicians require little to no additional consultation time [43]. Some additional processes—such as manually segmenting the ground truth—must still be performed by clinicians, but can be streamlined by a similar process; if a segmentation with series description “finalSegmentation” arrives in the PACS, the automatic data collection will detect this and associate it with the relevant series and study.
Data curation and cleaning	Data curation and cleaning are performed by experts or automatic processes, which are triggered manually.	Data curation (i.e. relevant series selection) is performed by automatic processes which are triggered as soon as the data arrives in the data lake. This can be performed with high accuracy based on ML methods using DICOM metadata [130, 131] or pixel data in images [132]. Data cleaning processes (i.e. conversion to inference-appropriate formats, application of pre-processing steps for data harmonisation) can be triggered following the appropriate data curation steps. Because D3C is automated, an appropriate informed consent waiver by the independent review board, guarantees that retrospective cohorts can be stored in the data lake, requiring only the identification of patients.
Model training	Models are trained on separate computers, which typically have no access to PACS systems or centralised data repositories.	Upon the selection of the relevant data for model training and internal validation, training can be triggered on local computational infrastructure or in the cloud.
Validation	Validation is performed after repeating D3C to gather additional sources of data, which are adequate for model validation. If continuous validation is necessary, this is typically performed at intervals by repeating this process.	Through automated D3C, both prospective and retrospective validation cohorts can be collected by simply identifying relevant patients or individuals. Validation can be triggered manually (which will run on pre-defined cohorts of patients) or orchestrated to run at specific intervals with data acquired recently—this is of paramount importance for continuous validation and monitoring of MedML models.
Model retraining	Model retraining requires repeating D3C with novel data. If retraining is intended with external data, the absence of a common protocol between hospitals or medical centres can cause additional complications, as these processes tend to be different between institutions.	Similarly to training, retraining with internal data requires only the selection of relevant patients or individuals. If training is to be done in a different centre with a standardised MedMLOps internal framework, this can be performed similarly after sharing of the data preprocessing and training protocol through containers, a technology which creates a reproducible environment and guarantees identical implementations between different medical institutions [133].

*PACS* picture archiving and communication system, *DICOM* digital imaging and communications in medicine, *D3C* data collection, curation and cleaning, *MedML* medical machine-learning, *MedMLOps* medical machine-learning operations

APIs simplify software interaction through standardised interfaces. Commands or sets of commands can be defined as scripts or algorithms, creating reproducible workflows. However, there can be multiple APIs, creating problems when one is updated or becomes obsolete and requires replacement. This leads to wasted time and resources for hospital IT staff. Furthermore, rapidly evolving development ecosystems can result in technical debt, with suboptimal implementations preventing maintenance and updates [99]. The development and/or use of consistent standards and their integration into MedMLOps systems solves this. Currently, some solutions for standardised EHR communication and interoperability are available [98] or for medical images [100–102]. Similarly, some MLOps platforms offer model serving standards with “model signature”, specifying expected inputs and outputs [103]. However, some aspects are still missing. Particularly, prediction from radiological images may depend on different sequences and clinical variables, requiring distinct levels of pre-processing. Importantly, models and medical data should operate under the same general systematic input and output specifications.

To remediate this, data models can be helpful. A data model relates different data objects [104] (medical images, clinical information, predictions). Data models could be built on top of PACS systems through existing standards such as Digital Imaging and Communications in Medicine (DICOM). These standards could then be used to create a systematic way of preparing data for prediction and for prediction storage and retrieval. Model inference would then require only the specification and retrieval of specific data formats. Data models are not new in medical data management [105]. Indeed, over 25,315 medical data models are available in the MDM Portal as of August 6th 2024^3^. However, depend on different institutions and follow different data storage and reporting practices. To unify data models, efforts like Prostate Imaging Reporting and Data System [106] or Breast Imaging Reporting and Data System [107]—uniform reporting standards for prostate multiparametric MRI and mammography, respectively—are helpful examples.

Commitment to solutions which are implemented but suboptimal when compared with modern solutions is common. Humans value products they already own and are less likely to switch to better ones if they have heavily invested in what they own (due to endowment effects and sunk-cost bias, respectively) [108, 109]. Organisations can be affected by organisational inertia, reducing their ability to internally change and innovate in the face of external changes [110, 111]. Reducing switching costs between MedML alternatives will maximise market benefits for stakeholders. MLOps can enforce standardised MedML system interactions, ensuring consistent interfaces for end users. This facilitates switching between MedML providers for developers, offloading complex system design to pre-specified standards.

Finally, regarding usability, user experience (UX) is important for clinicians as end users. UX guidelines in medical software are available [112], but studies focusing on UX for general clinical systems show that this can be improved [113]. Nonetheless, recent works have studied UX in computationally assisted diagnosis methods. For instance, less experienced users preferred using fully automated software programmes, while experts preferred a more step-by-step process [114]. MedML UX is a young field with ample grounds for growth as the number of deployed MedML products increases.

## Discussion: MedMLOps in context

We introduced MedMLOps as a concept, but note that others have considered and identified the necessity for MLOps in healthcare. For instance, Wiesenfeld and others discuss how MLOps can address issues pertaining to model transferability in the clinic and between healthcare centres [115]. Others have introduced MLOps frameworks. CyclOps contemplates data standardisation and validation of MedML models [116]. Resilience-aware MLOps focuses on resistance to adversarial attacks and domain drifts as key components of healthcare MLOps applications [117]. FlowEHR focuses on continuous validation, standardisation and expert supervision [118]. In a preprint, Khattak and others introduced Machine Learning for Healthcare Applications (MLHOps) as a technical framework focusing on standardisation, interoperability, trust, and bias reduction [119]. Importantly, recent multi-society position papers highlighted the need for continuously validating models to understand how performance changes, and continuously/locally training models to better serve different patient populations [119, 120]. MedMLOps facilitates this while reducing the burden of retraining and validating models from both clinicians and future MedML developers. The existing literature is not at odds with MedMLOps and is complementary to this work. However, we note that each of the pillars described above can be further expanded.

As noted, implementing MLOps systems is not straightforward. Considering current MedML products and systems, problems can arise in terms of data harmonisation, computational infrastructure and technical know-how. Concerning harmonisation, MedML systems lack standardised inputs and outputs, and may not work systematically across imaging conditions or scanners [14, 36, 37]. Medical institutions must acquire computational resources (cloud-based or local) and train/hire staff skilled in developing and maintaining MedML and MedMLOps. This expanding professional network will strengthen the MedMLOps ecosystem, assuring the ongoing safety of MedML systems.

Retraining may further hinder the implementation of MedMLOps. This is, in part, still uncertain from a regulatory perspective within the EU. The EUAI guarantees this is a possibility in high-risk applications if model and data documentation are adequately updated [121]. However, how this works from a regulatory perspective is still unclear, leading to added financial risk. According to the Medical Device Regulation, software devices (i.e. MedML models) can be retrained as long as this (i) does not significantly alter the performance, intended use or risk profile, (ii) follows the quality management protocols and post-market surveillance specified for the approval of the model and iii) is well-documented [122–124]. However, what constitutes significant alterations is not clear.

Lastly, we note that other actions can further improve MedML application use in the clinic. Education plans—both for medical doctors [26, 31, 70, 122] and patients [123–125]—are crucial to empower professionals. They ensure everyone is knowledgeable about the process and that patients can provide informed consent. Presently, the public is still distrustful of clinical AI applications. Levels of distrust are higher in disenfranchised people (lower educational attainment, non-Western immigrants, women) [126]. While understandable—there is a history of medical treatments discriminating against women, racialised individuals and those belonging to a lower socioeconomic status [127–132]—this can further create disparities and prevent a truly universal access to healthcare. Democratising MedML access should also consider the global stage: clinical trials for AI/MedML products are geographically concentrated in Europe, Asia and North America [133]. MedMLOps may not solve these issues entirely. However, universal frameworks for MedML deployment reduce entry barriers in disadvantaged regions and markets. Regions lacking specialised personnel and resources may benefit the most from high-quality and widely available MedMLOps systems.

## Conclusion

We define MedMLOps and how it can aid radiologists using MedML products, along with its challenges. These requirements expand MLOps to address healthcare-specific issues, achieved via communication between developers and medical personnel by systematising model implementation in a structured and automated manner.

## Abbreviations

AI: Artificial intelligence
API: Application programming interface
DevOps: Software development operations
DICOM: Digital imaging and communications in medicine
DTM: Data-to-model
EUAI: EU Artificial Intelligence Act
GDPR: General Data Protection Regulation
LLM: Large language model
MedML: Medical machine-learning (models)
MedMLOps: Medical machine-learning operations
ML: Machine-learning
MLOps: Machine-learning operations
MTD: Model-to-data
PACS: Picture archiving and communication system
UX: User experience

## Footnotes

1 Readers familiar with literature on reliable computer systems are likely to find some parallels with the concept of reliability, availability and serviceability (RAS) [50]; we note that our own selection of MedMLOps desiderata tried to make evident these three principles—availability ensures availability, continuous monitoring, validation and (re)training ensures reliability, and ease of use (for both the end-user and the developer) ensures serviceability.

2 https://kubernetes.io/

3 Accessible in: https://medical-data-models.org/
